# Reviewer acknowledgement 2012

**DOI:** 10.1186/2047-2994-2-4

**Published:** 2013-04-12

**Authors:** Andreas Voss

**Affiliations:** 1 Canisius-Wilhelmina Hospital and Radboud University Nijmegen Medical Centre, Netherlands

## Abstract

**Contributing reviewers:**

*Antimicrobial Resistance and Infection Control* would like to thank the following colleagues for their assistance with peer review of manuscripts for the journal in 2012.

## 

Hanan Balkhy

Saudi Arabia

Marc Bonten

Netherlands

John M. Boyce

United States of America

Peter Collignon

Australia

Yohei Doi

United States of America

Philippe Eggimann

Switzerland

Alexander W. Friedrich

Netherlands

Abdul Ghafur

India

Armando Gonzalez-Ruiz

United Kingdom

M. Lindsay Grayson

Australia

Mohan Gurjar

India

Joost Hopman

Netherlands

Jim Hutchinson

Canada

Vincent Jarlier

France

Estelle Jumas-Bilak

France

Drosos Karageorgopoulos

Greece

Keith Kaye

United States of America

Jan Kluytmans

Netherlands

Wolfgang Lindner

Germany

Jean-Christophe Lucet

France

Geeta Mehta

India

Shaheen Mehtar

South Africa

Leonard Mermel

United States of America

Christine Micolaud

France

Dilip Nathwani

United Kingdom

Eli Perencevich

United States of America

Didier Pittet

Switzerland

Celine Pulcini

France

Rosana Richtmann

Brazil

Thomas Riley

Australia

Helio Sader

United States of America

Jeroen Schouten

Netherlands

Mitchell Schwaber

Israel

Tom Sprong

Netherlands

Francois Stephan

France

Ellen Stobberingh

Netherlands

Paul Tambyah

Singapore

Jean-Francois Timsit

France

Athanassios Tsakris

Greece

Brigitte van Cleef

Netherlands

Henri Verbrugh

Netherlands

Margreet Vos

Netherlands

Joanne Wai-Yee Chung

Hong Kong

Andreas Widmer

Switzerland

Ina Willemsen

Netherlands

Mireille Wulf

Netherlands

Cordt Zollfrank

Germany

